# Establishing Transgenic Schistosomes

**DOI:** 10.1371/journal.pntd.0001230

**Published:** 2011-08-30

**Authors:** Victoria H. Mann, Sutas Suttiprapa, Gabriel Rinaldi, Paul J. Brindley

**Affiliations:** 1 Department of Microbiology, Immunology & Tropical Medicine, George Washington University Medical Center, Washington, D.C., United States of America; 2 Departamento de Genética, Facultad de Medicina, Universidad de la República, (UDELAR), Montevideo, Uruguay; Washington University School of Medicine, United States of America

Draft genome sequences for *Schistosoma japonicum* and *Schistosoma mansoni* are now available. The schistosome genome encodes approximately 13,000 protein encoding genes for which the function of only a small minority is understood. The new genes represent potential intervention targets. Molecular tools are needed to determine the importance of these new genes. There is a role for transgenesis in functional genomics of the new schistosome gene sequences, both in gain- and loss-of-function approaches such as insertional mutagenesis screens and vector-based RNA interference. This laboratory symposium focuses on the development of approaches, systems, and tools to address the problem of establishing transgenic schistosomes which, in turn, can be applied to fundamental questions of schistosome physiology, the host–parasite relationship, and to developing new interventions.

## The Problem

Control of schistosomiasis largely relies on chemotherapy, but people rapidly become re-infected and the widespread use of praziquantel has led to concerns about development of drug resistance. Advances in molecular genetics, biochemistry, and vaccinology hold promise to control the spread of schistosomiasis and to combat the morbidity and mortality associated with this neglected tropical disease. Draft genome sequences for *Schistosoma japonicum* and *Schistosoma mansoni* are available [1], [2]. Molecular tools are needed to determine the importance of newly identified genes. Problematically, few functional genomics tools are available for schistosomes. The potential value of transgenesis approaches for schistosomes is obvious given the progress made in model species and cell lines and indeed more tractable pathogenic species (e.g., [3]–[5]). There is a valuable role for transgenesis in functional genomics for investigation of schistosome genes. Devising tools to create transgenic schistosomes and deploying transgenic schistosomes in functional genomics analysis will advance knowledge of schistosomes and schistosomiasis.

## Tutorial

### Why Pursue Transgenesis for Schistosomes?

Transgenesis, including somatic and germ line approaches, is a desirable goal. It is a well-established approach for functional genomics in model species including *Caenorhabditis elegans* and *Drosophila melanogaster* (e.g., [6]). It should be able to facilitate gain-of-function and/or loss-of-function phenotypic and molecular analysis in schistosome parasites. Transgenesis approaches can facilitate vector-based RNA interference, and would be a potential forward genetic technology for insertional mutagenesis screens, which are feasible now that draft schistosome genome sequences are available. In addition, transgenes are potential tools for development of genetic therapy and/or vaccines. Approaches being developed for schistosome transgenesis include deployment of integration-competent vectors such as transposons and retrovirus. Integration-competent vectors are expected to lead to insertion of transgenes into schistosome chromosomes.

### Which Vectors Can Be Used to Produce Transgenic Schistosomes?

Both integration-competent and non-integrating plasmids have been used to introduce transgenes into schistosomes [7]–[9]. Although both approaches have utility, there are compelling reasons to focus on integration-competent vectors, primarily because integrated transgenes can be propagated equally and reliably to the progeny of the transduced cell, including germ line cells. Integration-competent vectors include DNA transposons such as *marine*r, *Sleeping Beauty*, and *piggyBac*, and simple and complex retroviruses including murine leukemia viruses and lentiviruses such as HIV-1. Indeed, colleagues in our lab have demonstrated the proof of this principle by showing that the transposon *piggyBac* is transpositionally active in *S. mansoni* [8], and the vesicular stomatitis virus glycoprotein (VSVG)-pseudotyped murine leukemia retrovirus (MLV) can transduce *S. mansoni* and *S. japonicum*, leading to active proviral reporter transgenes integrated in the schistosome chromosomes [9]–[11]. Other approaches including deployment of bacteriophage integrases and fungal recombinases, which have found service in genome manipulation of, for example, *Plasmodium falciparum*, may also be of use [12], [13], but have not yet been reported with schistosomes.

### Which Developmental Stages Might Be Targeted?

**Figure 1:**
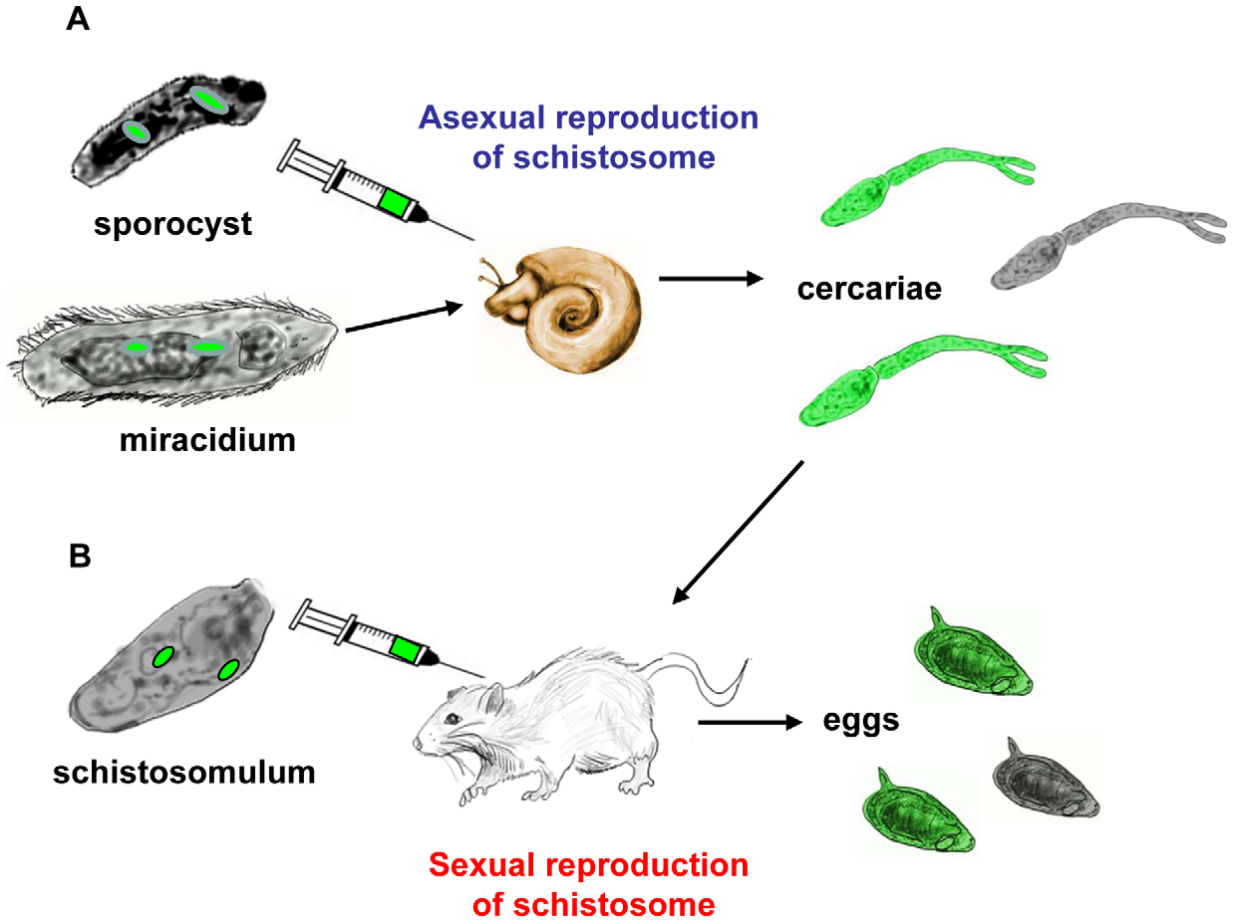
Schematic representation of general strategies that can be explored to introduce transgenes into the germ line involving the asexual (A) and sexual (B) reproduction processes of the developmental cycle of *S. mansoni*. In brief, eggs/miracidia, sporocysts, and/or schistosomula might be transduced by VSVG-pseudotyped Moloney murine leukemia retrovirus. Subsequently, snails can be infected with miracidia by the natural percutaneous route or with sporocysts by microinjection and mice infected by the parenteral route with transformed schistosomules or by the natural percutaneous route by cercariae. Progeny cercariae from snails and eggs from mice can be analyzed for transgenes and/or reporter transgene activities. The green colored cells in illustrations of larvae represent the potential presence of transgenes in germ line and/or somatic cells.

Theoretically, the schistosome genome is targetable at any stage of parasite development given that for *S. mansoni*, for instance, the entire developmental cycle can be maintained in the laboratory in *Biomphalaria* species snails and the laboratory mouse (Figure 1). Some stages can be cultured ex vivo or in vitro, and returned to the snails or mice to continue development (see [14]). Other stages have potential advantages as targets for transgenes given their accessibility, tolerance to manipulation, size, and/or ratio of germ to soma (e.g., [10], [15]). Also, schistosome stages are differentially accessible to delivery of transgenes, using approaches that have included particle bombardment, square wave electroporation, cationic polymer-based gene delivery, and infection of schistosomes with pseudotyped retrovirus [7], [16], [17]. Other approaches, including microinjection, should be of value, as indicated by progress with introduction of transgenes in parasitic nematodes [18]. The schistosome egg and the miracidium that hatches from the mature egg have desirable attributes for consideration in relation to transgenesis. These include the presence of the single cell zygote within the eggshell upon its release from the female blood fluke, high ratio of germ to somatic cells, ease of maintenance in vitro, accessibility of embryonic cells within the egg to transgenes of pseudotyped retrovirus virions, and ability of the miracidium, which is readily released from the mature egg by transfer of the cultured egg into sterile water to naturally infect the intermediate host snail [19], [20]. The attributes also include availability of eggs from livers of experimentally infected rodents [7], [14] and ability of the female to deposit viable eggs in vitro [21] (see below). Moreover, from a clinical perspective, the egg represents the major source of pathogenesis. Figure 1 outlines strategies that can explored to direct transgenes to the germ line of *S. mansoni* involving the asexual and sexual reproduction processes of the developmental cycle of the parasite.

#### Eggs and miracidia

In the first report of vertical or germ line transgenesis in schistosomes, Grevelding and colleagues [7] transfected miracidia with plasmid DNA encoding green fluorescent protein (GFP) driven by the schistosome actin gene promoter, and reported that the GFP transgene was transmitted to the F1 generation of miracidia, via passage through snails and mice. Transmission to the F2 and F3 generations was not apparent, though, likely because the transgene transmission as episomal and the extra-chromosomal transgene was diluted and/or lost in subsequent development and generations. Non-integrated, extra-chromosomal arrays of plasmid transgenes are the normal occurrence in transgenic *C. elegans* where transgene DNA assembles through non-homologous recombination into multi-copy concatemers or extra chromosomal arrays. These are inherited in non-Mendelian fashion. Nonetheless, integration into the *C. elegans* genome will improve transmission to the progeny (see [22]). Recently, we demonstrated the feasibility of manipulating eggs of *S. mansoni*. Eggs from mouse livers were soaked and/or electroporated with different reporter transgenes including MLV virions. Eggs were transduced with virions after which retroviral transgenes were detected and quantified in the genome of miracidia by real-time PCR [20].

#### In vitro laid eggs (IVLE)

**Figure 2:**
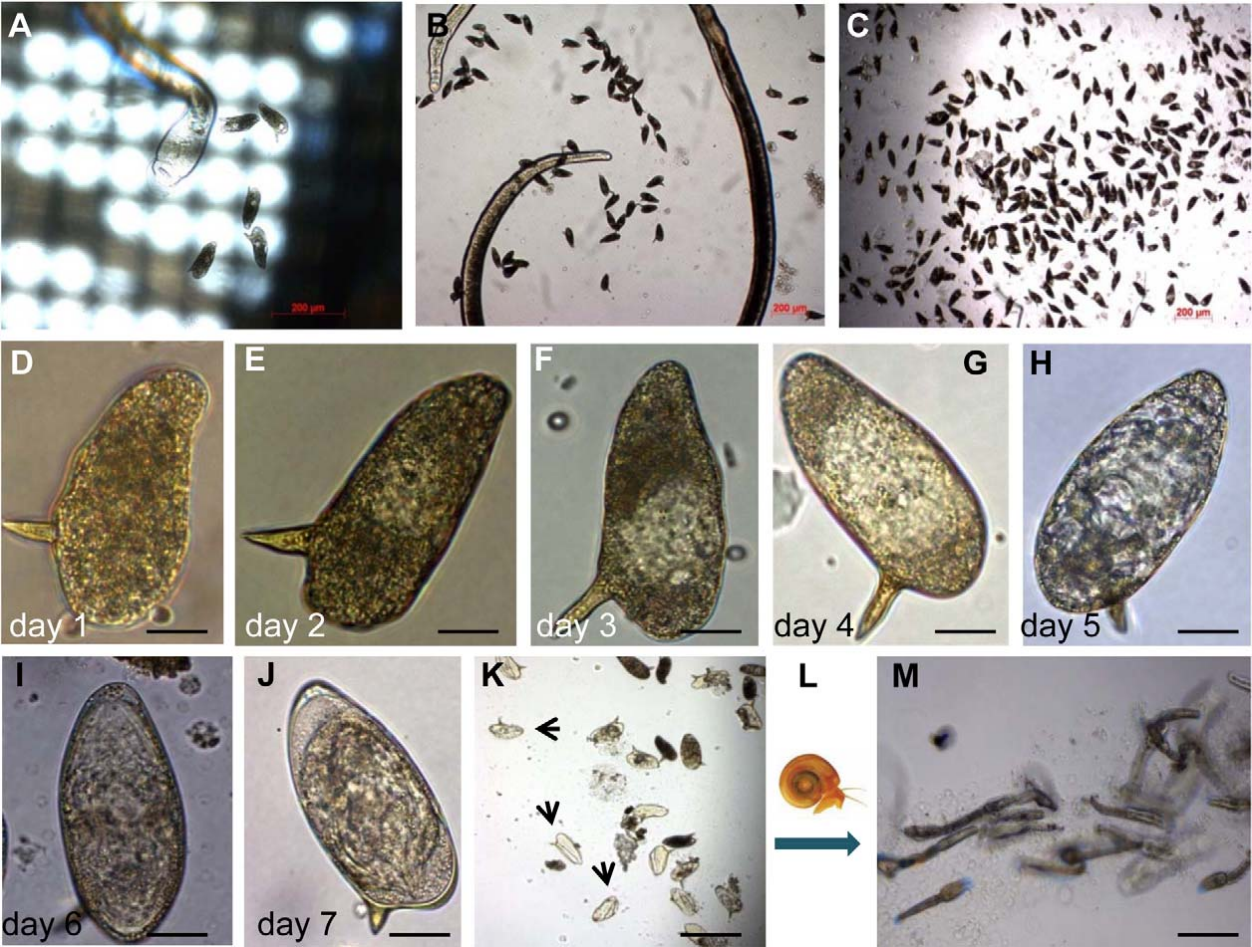
Representative pictures of the in vitro laid eggs (IVLE) collection, concentration, and in vitro development. (A) Female of *S. mansoni* releasing eggs one day after perfusion. The mesh of the netwell is evident. (B) Female surrounded by IVLE in the bottom of the well. (C) IVLE during the concentration process 48 hours after perfusion. (D–J) Representative images of an individual egg laid in vitro through the developmental process from day 1 (D), 2 (E), 3 (F), 4 (G), 5 (H), and 6 (I) to day 7 (J) after perfusion. (K) IVLE during the hatching process; arrows indicate empty eggshells. (L) Diagram of snail infection. (M) Cercariae released from snails infected with miracidia from IVLE, at 42 days after snail infection. Scale bars: (A–C,K,M), 200 µm; (D–J), 20 µm.

We have begun to focus on eggs laid in vitro by females aiming to target transgenes to the zygote or to the early blastula where the total number of cells would be less than in the mature egg and where the germ to somatic cell ratio would be higher, which would enhance the likelihood of transfecting germ cell(s), a prerequisite for perpetuating an entirely transgenic schistosome or a mosaic form comprising transgenic and non-transgenic tissues. One approach to this goal involves culturing schistosomes recovered from mice and collection of eggs laid in vitro by these worms within 48 hours. (Eggs laid after the females have been in culture for >48 hours do not develop correctly [21].) With these in vitro laid eggs (we abbreviate as IVLE), we follow the protocol of Mann et al. [14], with modifications. We transfer adults into schistosomule medium and maintain them at 37°C immediately after perfusion from mice, and transfer the worms as soon as practicable into 74-µm diameter mesh netwell, 6-well plates (Fisher Bioscience, catalog no. 07200213). The worms are maintained in schistosomule medium in netwell plates for 48 hours after perfusion. IVLE and, occasionally, adult females fall through the mesh and collect on the bottom of the culture plate (Figure 2A). At 48 hours after perfusion of the worms from mice, we collect IVLE, and concentrate by filtering media containing IVLE through 0.8-µm mesh transwell (BD Biosciences, catalog no. 353097) (Figure 2B, 2C). Thereafter, IVLE are maintained in schistosomule medium where they develop and mature within 7 days (Figure 2D–2J). At that point, we transferred IVLE to sterile water, illuminated the culture with a bright lamp, and observed that many eggs (30% to 40%) hatch within 120 minutes. We observed that miracidia hatched from IVLE infected *Biomphalaria glabrata* snails from which, in turn, cercariae were released after 6 weeks (Figure 2K–2M). IVLE represents a tractable, developmental stage at which to target transgenes, especially since in developmental stages 0 and 1 (staging system of Jurberg et al. [19]), no cleavage of the zygote cell has yet taken place. In future studies, we plan to expose IVLE to pseudotyped MLV virions at the time eggs are released from the female schistosome, aiming to introduce transgenes into the schistosome germ line. (The studies with schistosome-infected laboratory mice were undertaken with the approval of the IACUC of The George Washington University, Washington, D.C.).

### How Can We Increase the Likelihood of Chromosomal Integration?

For transposons, in particular for binary versions of broad host range vectors such as *piggyBac* and *Sleeping Beauty* (e.g., [4], [8]), increased efficiency of integration can be accomplished using mRNA encoding the transposase rather than using helper plasmid, and further, optimal ration of transposon and transposase can be titrated. Also, transposase enzyme can be employed instead of mRNA of the gene [3]. For retroviruses, in particular MLV, with which we have some experience, increasing the titer of active virions is a sound way to improve prospects for productive transduction of target germ line cells. Given the progress with transgenesis of schistosomes with pseudotyped MLV [9]–[11], [20], [23], [24], comments on virion production are included below.

#### Can virion production be optimized?

**Figure 3:**
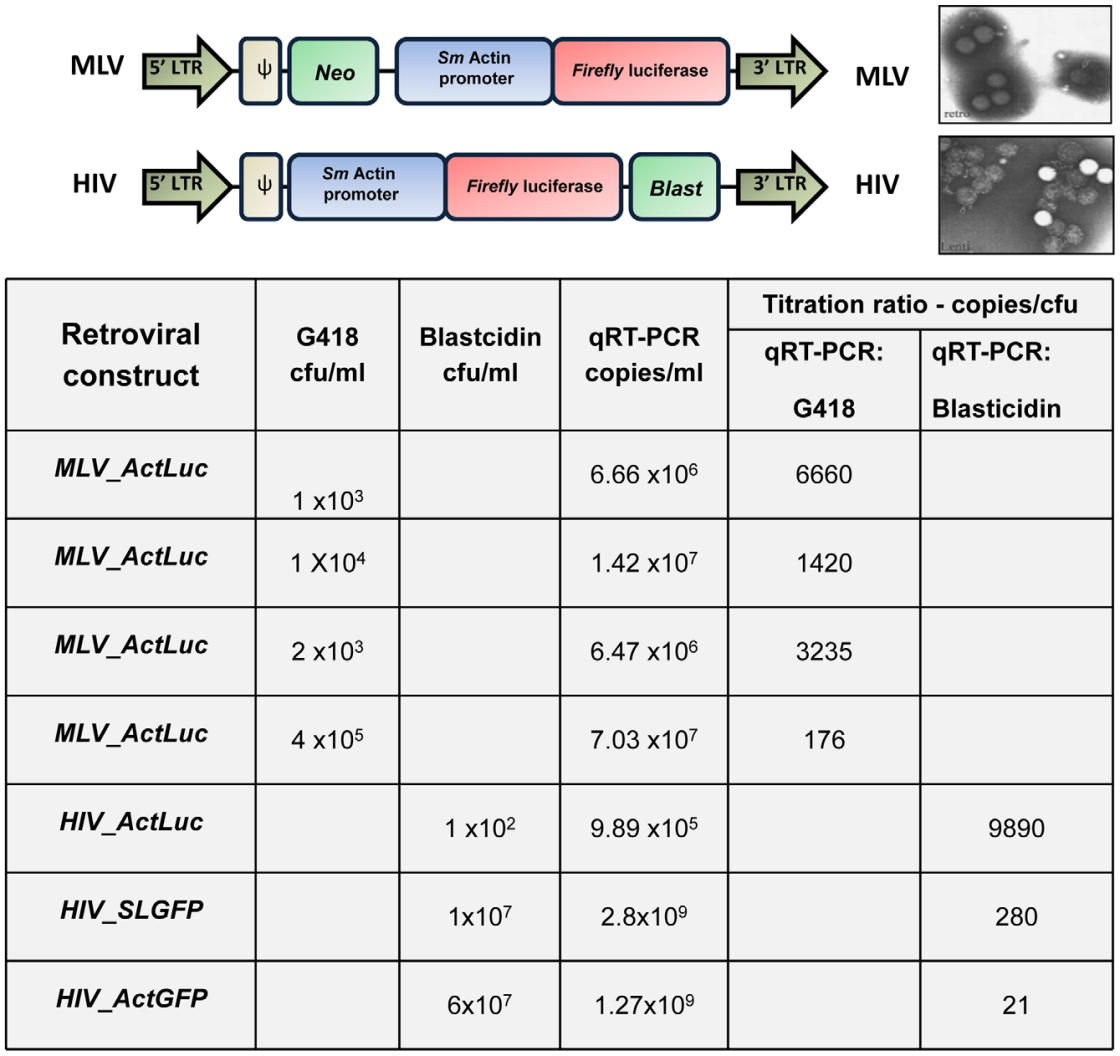
Correlating RNA titer with infectivity. Schematic diagram of two MLV and HIV representative constructs and corresponding micrographs of the particles (from Higashikawa et al. [25], with permission). To determine infectivity titers, NIH 3T3 or HT1080 cells were infected with qRT-PCR-titrated MLV retrovirus or qRT-PCR-titrated HIV, carrying neomycin (*Neo*) or blasticidin (*Blast*) resistance genes, respectively. Cells were selected in G418 or blasticidin for 10 days and resistant colonies were stained and counted.

There are several factors to consider for an optimal virus production, including producer (host) cell strain and culture conditions, cell density and vitality by the time of the DNA transfection, amount and quality of plasmid DNA used for transfection, and recovery of viral particles. Factors that reduce the retrovirus half-life also should be considered, i.e., storage of virions, freeze-and-thaw cycles, temperature, pH, and presence of serum [25]. The higher the viral titer, the better the prospects are for chromosomal insertion of the proviral transgene [26]. Although protocols to produce retrovirus in vitro optimize to improve titers, contamination of virions with defective particles is a frequent problem. Defective virions include particles without envelope, without RNA, or with RNA but non-infectious [27]. Using at least two approaches to estimate viral titer is recommended [25], [27]. One approach should estimate the titer of infectious particles, i.e., a functional, biological assay where a cell line is infected with serial dilution of the virions and colonies of cells are selected by maintenance in antibiotic for which resistance is conferred by the retrovirus. Second, a quantitative approach to estimate the copy number of particles by qPCR should also be performed in parallel. With findings from the parallel assay, total number of virions particles and of intact, infectious particles can be established [25], [27]. We use both approaches to estimate the viral titer and the ratio between copy number of particles and infectious units [24]. Figure 3 presents representative findings from our laboratory, for both MLV and HIV pseudotyped virions; in general, titers estimated by qPCR were two to three orders of magnitude greater than those estimated in biological assays. This is not only because qPCR is more sensitive, but also because qPCR detects non infective particles. Accordingly, the lower the titration ratio, the more efficient is the virus production in terms of viable infectious particles.

### How Can We Analyze Integration of Transgenes into the Genome?

**Figure 4:**
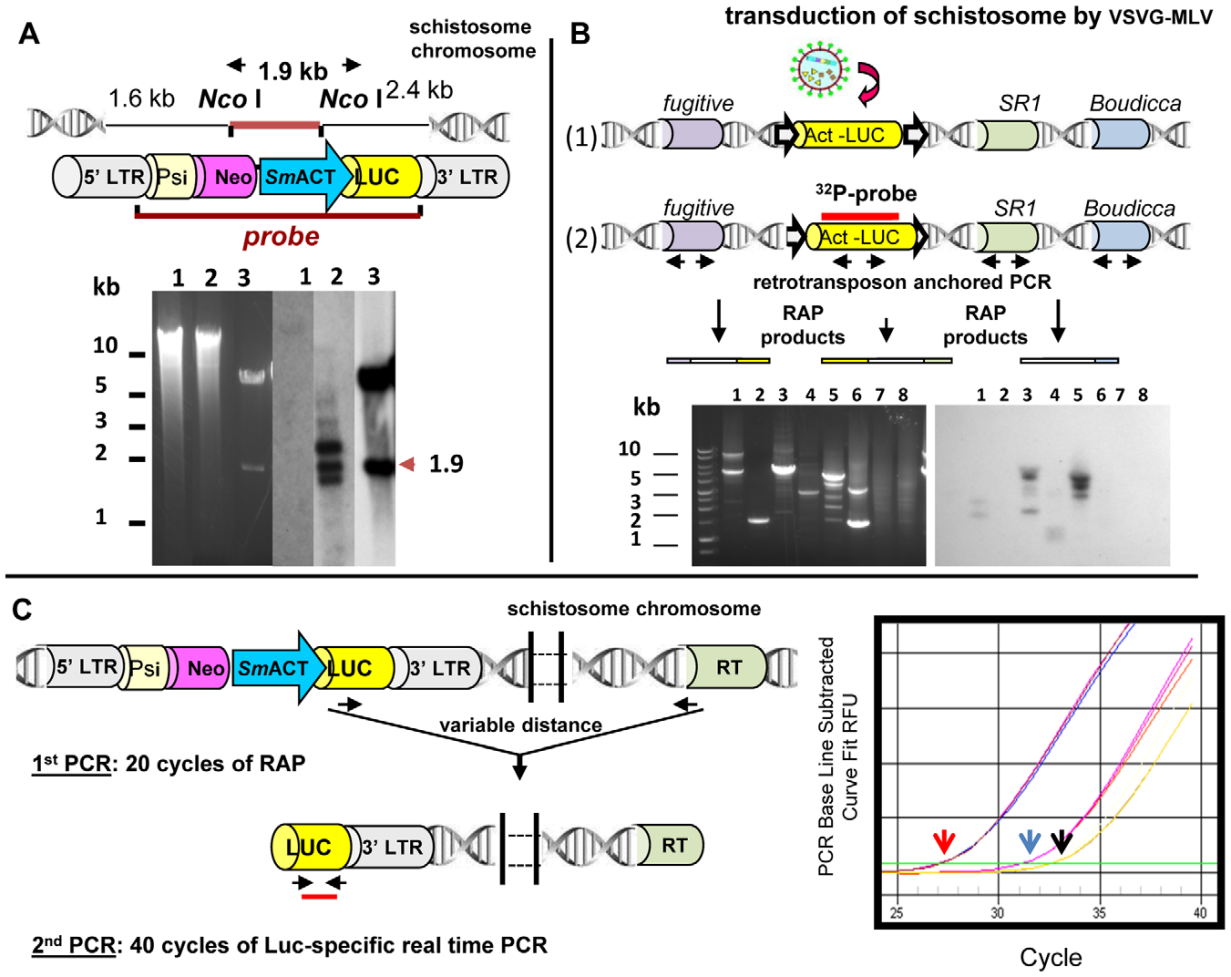
Approaches to identify, clone, and quantify integrated sequences into the schistosome genome. (A) Integration of retroviral provirus into the *S. mansoni* genome indicated by Southern hybridization analyses of genomic DNA from retrovirus-transduced schistosomes. *Top Panel*: Schematic representation of retroviral construct pLNHX-*Sm*ACT-Luc, showing the position of *Nco* I cleavage sites and also the location of the *Kpn* I fragment employed as the hybridization probe. The retrovirus cassette included the firefly luciferase reporter gene (yellow) driven by the *S. mansoni* actin 1.1 gene promoter (blue), and flanked by the 5′- and 3′-long terminal inverted repeats of the murine leukemia virus (grey). The cassette also included the gene endowing neomycin resistance (pink) and the psi motif (light yellow; involved in packaging the viral DNA). *Bottom Panel*: Southern hybridization analysis of genomic DNA from schistosomules transduced by VSVG-pseudotyped retrovirus. Left side: ethidium-stained gel of genomic DNAs of *S. mansoni*: lane 1, *Nco* I-digested gDNA from control, non-virus transduced cercariae; lane 2, *Nco* I-digested gDNA from schistosomules exposed to VSVG-pseudotyped pLNHX-*Sm*ACT-Luc virions; lane 3, *Nco* I-digested plasmid pLNHX-*Sm*ACT-Luc. Molecular size standards in kilobases (kb) shown at margin. Right side: autoradiograph of Southern hybridization signals from the *Nco* I-digested gDNAs and plasmid DNA from the left-side panel to the radiolabeled transgene probe (5.3 kb *Kpn* I fragment of pLNHX-*Sm*ACT-Luc, top panel). (Modified from [9] with permission.) (B) *Top Panel:* Schematic representation of the RAP technique, designed to recover integration junctions between integrated retroviral provirus and endogenous mobile genetic elements resident within the *S. mansoni* genome. (1) Schematic representation of the integration of the MLV retrovirus into schistosome chromosomes after transduction of cultured schistosomes by VSVG-pseudotyped MLV virions. Endogenous retrotransposons within the schistosome chromosomes are shown; numerous copies of *SR1*, *Boudicca*, and the *fugitive* have been described interspersed throughout the *S. mansoni* genome. (2) Schematic depiction of the RAP technique used to investigate transgene integrations. The position of the primers used in the PCRs and the probe used in Southern hybridizations is indicated. *Bottom Panel:* Left side. Ethidium-stained gels revealing RAP products amplified from gDNA extracted from schistosomula transduced with VSVG-pseudotyped pLNHX-SmACT-Luc virions; the PCR products were amplified using primers specific for the endogenous schistosome retrotransposons and the luciferase transgene. Right side: Southern hybridization of labeled retroviral transgene gene probe to the RAP products shown in top panel. Lane 1, RAP products amplified with luciferase left and *Boudicca* forward-directed primers; lane 2, RAP products from luciferase left- and *fugitive* forward-directed specific primers; lane 3, RAP products from luciferase left- and *fugitive* reverse-directed specific primers; lane 4, RAP products from luciferase left and *SR1* forward-specific primers; lane 5, RAP products from luciferase left- and *SR1* reverse-specific primers; lane 6, RAP products from luciferase left- and *SR2* forward-specific primers; lane 7, RAP products from luciferase left- and *SR2* reverse-specific primers; lane 8, RAP products from luciferase left- and SMα forward-specific primers. Values at left are molecular size standards (kb). (Modified from Kines et al. [9], with permission) (C) *Left Panel:* Illustration of the quantitative retrotransposon anchored PCR (qRAP). Schematic representation of the integration of the MLV retrovirus into schistosome chromosomes after transduction of cultured schistosomes by VSVG-pseudotyped MLV virions. First PCR: 20 cycles of end-point PCR preamplification with primers that target endogenous mobile genetic elements and luciferase transgene sequences. Heterogeneous amplicons of variable length are expected. Second PCR: quantitative PCR to estimate the copy number of luciferase-specific sequences within the transduced schistosome genome. Quantification was undertaken using copy number standards, i.e., 10-fold serial dilutions of the luciferase encoding plasmid pGL3, after which copy number of luciferase transgene in schistosome genomic DNAs was calculated by interpolation from a standard curve. *Right Panel:* Amplification plots observed in MLV-transduced worms in preamplified template using *SR2* primer mix (red arrow), in preamplified template using only the luciferase transgene–specific primer as control of “one-way amplification” (blue arrow), and in non-preamplified template (black arrow). Arrows indicate the threshold cycle. RT: endogenous retrotransposon. (Modified from Rinaldi et al. [24], with permission.)

Integration is a pivotal step in establishing transgenic schistosomes. To confirm that vectors can integrate into schistosome DNA, methods to determine and extract integration junctions have to be employed. Integration junctions cannot be obtained simply by regular PCR procedures because the genomic flanking sequences are unknown. Southern hybridization employing informative restriction enzymes and probes retain a key position in these studies. For example, Figure 4A presents a Southern hybridization analysis to confirm the presence of proviral transgenes in the genome of schistosomules exposed to pseudotyped MLV virions. However, the definitive proof of integration of the transgenes into the schistosome chromosome requires the use of PCR-based approaches directed at cloning and sequencing the integration junction (Figure 4B). Given that draft genomes of *S. mansoni* and *S. japonicum* are available, BLAST analysis of cloned sequences flanking the transgenes (e.g., MLV retrovirus) can readily verify that the transgenes have integrated into a schistosome chromosome. A number of PCR-based methods can be employed to recover integration events and unknown host genome sequences flanking the transgene. These include inverse PCR, linker ligation PCR and thermal asymmetric interlaced (TAIL)-PCR, and *Alu*-PCR [28], [29]. We developed an *Alu*-PCR-like approach termed retrotransposon anchored PCR (RAP) [8], [9], which relies on anchoring primers to multi-copy endogenous mobile genetic elements interspersed in the schistosome genome to locate integration junctions of transgenes in the genome of *S. mansoni* (Figure 4B). Furthermore, we have adapted RAP for quantitative PCR in order to determine comparatively the number of MLV transgenes within the genome of transduced populations of schistosomes (Figure 4C) [24]. In addition, very high-throughput sequencing using Illumina technology could be utilized, as has been demonstrated for transposon-based insertional mutagenesis of *Salmonella* Typhi [3]. This latter approach could determine the exact location of transgenes within the schistosome genome.

## Outlook

As noted, transgenic schistosomes have been created (e.g., [7]–[9], [23]). However, improvements are needed—and certain to take place—in order to establish transgenic schistosomes and protocols. A crucial impediment to date has been the difficulty of delivering transgenes to chromosomes of the germ line. Targeting integration-competent vectors to IVLE may surmount this limitation. Moreover, there are other points in the developmental cycle where the germ line might be accessed, including the daughter sporocysts where the germ cells are comparatively large (Coustau and Yoshino [30] and references therein). We can look forward to advances in technologies that will drive functional genomics forward quickly, including expansion of in vivo RNAi, high-throughput insertional mutagenesis and, hopefully, gains-of-function approaches involving drug selection of transgenic schistosomes. Advances in *S. mansoni* can be expected to be adapted to the other schistosomes, to the food-borne flukes such as *Fasciola* and *Opisthorchis* species, and to neglected helminth parasites at large.

**Key Learning Points**

- Draft genome sequences for *S. mansoni* and *S. japonicum* are now available. Accordingly, there is a pressing need now to develop functional genomics tools for schistosomes to determine the importance of these new genes.
- Functional genomics approaches hold promise to determine the nature and importance of genes of the human schistosomes.
- Retroviral and transposon-mediated gene manipulation, using integration-competent, vector-based technologies, have been shown to be feasible for schistosomes.
- The retrovirus murine leukemia virus, which is widely used in stem cell and gene therapy research, and the *piggyBac* transposon, originally isolated from the genome of a moth, have now been shown to be active in schistosomes, to integrate into schistosome chromosomes, and to provide gains-of-function for the reporter genes firefly luciferase, GFP, and neoR.
- Both MLV and *piggyBac* both have potential in high-throughput insertional mutagenesis studies, feasible now that draft genome sequences are available.
- Vector-based RNAi—retroviral vector-mediated RNA interference demonstrated in schistosomes—targeting a hemoglobin-digesting protease provides proof-of-principle that vector-based RNAi is feasible to target any of the ∼13,000 protein encoding genes of the schistosome.

## Supporting Information

Alternative Language Abstract S1 Translation of Abstract into Thai by Sutas Suttiprapa.
(PDF)

Alternative Language Abstract S2 Translation of Abstract into Spanish by Gabriel Rinaldi.
(PDF)
