# Supplementary material for: Establishing Transgenic Schistosomes
Source: PLoS Negl Trop Dis. 2011 Aug 30;5(8):e1230. doi: 10.1371/journal.pntd.0001230 (PMC3166048; doi:10.1371/journal.pntd.0001230)
Supplement: Alternative Language Abstract S1 — Translation of Abstract into Thai by Sutas Suttiprapa. (PDF) [file pntd.0001230.s001.pdf]

Alternative Language Abstract S1. Translation of Abstract into Thai by Sutas Suttiprapa.

การสร้างพยาธิ์ชีสโตโซมแปลงพันธุ์

Victoria H. Mann[1,*], Sutas Suttiprapa[1,*], Gabriel Rinaldi[1,2,*], Paul J. Brindley[1]

1 Department of Microbiology, Immunology & Tropical Medicine, George Washington University Medical Center, Washington, DC 20037 USA

2 Departamento de Genética, Facultad de Medicina, Universidad de la República, (UDELAR), Montevideo, Uruguay

* Equal contribution

บทคัดย่อ

พิมพ์เขียวของจีโนมของพยาธิ์ชีสโตโซม (schistosome)ชนิด Schistosoma japonicum และ S. mansoni ขณะนี้สามารถนำมาใช้ประโยชน์ได้แล้ว ลำดับดีเอ็นเอของพยาธิ์ชีสโตโซมประกอบด้วยยีนที่สามารถแปรรหัสเป็นโปรตีนได้ประมาณ 13,000 ชนิด ซึ่งมีส่วนน้อยของยีนเหล่านี้เท่านั้นที่ทราบบทบาทหน้าที่ในพยาธิ์ ยีนใหม่ที่ยังไม่ทราบหน้าที่เป็นเป้าหมายที่สำคัญในการพัฒนาการรักษา เครื่องมือในระดับโมเลกุลเป็นสิ่งที่ต้องการเพื่อใช้ตรวจหาความสำคัญของยีนใหม่ ๆ เหล่านี้ การแปลงพันธุ์ (transgenesis) มีบทบาทในการศึกษาความสำคัญของยีน โดยการศึกษาด้วยวิธีการเพิ่มและการลดบทบาทหน้าที่ของยีน เช่นการตรวจกรองผลของการกลายพันธุ์ (insertional mutagenesis screening) และการแทรกแซงการแสดงของยีนด้วยอาร์เอ็นเอ (RNA interference) ซึ่งบทความนี้จะเน้นการพัฒนาวิธีการ ระบบ และเครื่องมือเพื่อแสดงให้เห็นถึงปัญหาของการสร้างพยาธิ์ชีสโตโซมแปลงพันธุ์ ซึ่งสามารถนำไปประยุกต์ใช้ในการตอบปัญหาพื้นฐานทางด้านสรีรวิทยา ความสัมพันธ์ระหว่างโฮสต์กับพยาธิ์ และการพัฒนาการรักษาด้วยวิธีใหม่
