# Supplementary material for: Establishing Transgenic Schistosomes
Source: PLoS Negl Trop Dis. 2011 Aug 30;5(8):e1230. doi: 10.1371/journal.pntd.0001230 (PMC3166048; doi:10.1371/journal.pntd.0001230)
Supplement: Alternative Language Abstract S2 — Translation of Abstract into Spanish by Gabriel Rinaldi. (PDF) [file pntd.0001230.s002.pdf]

Alternative Language Abstract S2. Translation of Abstract into Spanish by Gabriel Rinaldi.

Generando schistosomas transgénicos.

Victoria H. Mann[1,*], Sutas Suttiprapa[1,*], Gabriel Rinaldi[1,2,*], Paul J. Brindley[1]

1 Department of Microbiology, Immunology & Tropical Medicine, George Washington University Medical Center, Washington, DC 20037 USA
2 Departamento de Genética, Facultad de Medicina, Universidad de la República, (UDELAR), Montevideo, Uruguay

* Idéntica contribución al trabajo

Resumen

Las secuencias completas de los genomas de *Schistosoma japonicum* y *S. mansoni* se encuentran actualmente disponibles. El genoma del schistosoma comprende ~13,000 genes codificantes de proteínas de los cuales solo se conoce la función de muy pocos. El desarrollo de herramientas moleculares es necesario para determinar la importancia de estos nuevos genes, muchos de los cuales representan blancos potenciales de intervención. En este sentido la transgénesis cumple un rol vital en la genómica funcional de estas nuevas secuencias génicas, tanto en estudios de ganancia como pérdida de función, donde la mutagénesis insercional a mediana y gran escala, así como la interferencia de ARN mediada por vectores de expresión son solo algunos ejemplos. Este simposio de laboratorio se centra en el desarrollo de dichas tecnologías, sistemas y herramientas para resolver el problema de establecer líneas de schistosomas transgénicos lo cual permitirá responder preguntas fundamentales de la fisiología de *Schistosoma*, la interrelación huésped-parasito y el desarrollo de nuevas estrategias de intervención y control parasitario.
